# Study on the Potential Mechanism of miR-22-5p in Non-Small-Cell Lung Cancer

**DOI:** 10.1155/2022/3750734

**Published:** 2022-09-06

**Authors:** Xuemei Han, Hua Li, Shuhui Liu, Zhiqiang Zhao

**Affiliations:** ^1^Department of Respiratory and Critical Care Medicine, Second Hospital of Tianjin Medical University, Tianjin 300211, China; ^2^Tianjin Key Laboratory of Ionic-Molecular Function of Cardiovascular Disease, Department of Cardiology, Tianjin Institute of Cardiology, Second Hospital of Tianjin Medical University, Tianjin 300211, China

## Abstract

**Objective:**

Non-small-cell lung cancer (NSCLC) ranks among one of the most lethal malignancies worldwide. A better and comprehensive understanding of the mechanism of its malignant progression will be helpful for clinical treating NSCLC.

**Methods:**

The miRNA expression profiles and target gene profiles downloaded from the Gene Expression Omnibus and TargetScan databases were used to identify the key regulatory pattern in NSCLC by bioinformatic analysis. The regulation of miRNA to target mRNA was verified by luciferase reporter assay, quantitative real-time polymerase chain reaction (qRT-PCR), and Western blot. A series of the in vitro and in vivo experiments were conducted to examine the mechanism of the overexpression or knockdown of the miRNA and/or target gene.

**Results:**

In this study, miR-22-5p was remarkably downregulated in NSCLC than in normal lung cells. The in vitro experiments showed that it could substantially inhibit NSCLC cell proliferation, invasion, migration, and epithelial–mesenchymal transition (EMT) progression. The results of luciferase reporter assay, qRT-PCR, and Western blot revealed that TWIST2 was a direct target gene of miR-22-5p. The results of in vitro and in vivo feedback experiments further demonstrated that miR-22-5p relied on TWIST2-induced malignant progression to regulate NSCLC proliferation, metastasis, and EMT progression.

**Conclusions:**

This study revealed that miR-22-5p downregulation contributed to the malignant progression of NSCLC by targeting TWIST2. The findings highlight a potential novel pathway that could be therapeutically targeted in treating NSCLC.

## 1. Introduction

Lung cancer ranks as the most lethal cause of cancer-related mortality for men and women worldwide [1], and non-small-cell lung cancer (NSCLC) accounts for more than 80% of all lung cancers [2]. Although a series of treatments for NSCLC are available, the mortality associated with NSCLC remains high because of metastasis, recurrence, or drug resistance caused by malignant progression [3–5]. Thus, more therapeutic strategies are needed. Elucidating the molecular mechanism of NSCLC's malignant progression is vital for the identification of new potential therapeutic targets.

Malignant progression is reflected in tumor proliferation and metastasis, which could be mediated by epithelial–mesenchymal transition (EMT) [6, 7]. The malignant progression of cancer is caused by the dysregulation of the expression of cancer-related genes, and the levels of these genes could be regulated by miRNAs [8, 9]. An increasing number of miRNAs have been found to regulate the malignant progression of multiple cancers [10]. Hence, the identification of the key regulatory miRNAs and the clarification of their regulatory mechanism will be helpful to better understand NSCLC.

In the present study, changes in the miRNAs between NSCLC tissues and adjacent normal tissues were compared for screening the key regulatory miRNA, and miR-22-5p was identified. A series of tumor types, such as liver cancer, gastric cancer, thyroid cancer, and breast cancer, exhibit a dysregulation of miR-22-5p [11–14]. However, whether and how miR-22-5p regulate the malignant progression of NSCLC are unclear and need to be assessed. In this study, we aimed to explore the regulatory mechanism of miR-22-5p in NSCLC and provide a biomarker of NSCLC to guide clinical medication, having important clinical application value.

## 2. Materials and Methods

### 2.1. Omics Analysis

The miRNA expression data in NSCLC and adjacent normal tissues used in this research were downloaded from the Gene Expression Omnibus (GEO) database (dataset GSE171517). This dataset includes six NSCLC tissues and corresponding adjacent normal tissues. Differentially expressed miRNAs were analyzed based on a |log2fold change| ≥ 1.8 using the *R* package of limma [15], and the targets of the miRNA were analyzed using the TargetScan database [16]. The target screening was based on the cumulative weighted context score and the potential function mechanism of miRNA.

### 2.2. Cell Culture and Transfection

Human normal lung cell line (BEAS-2B) and NSCLC cell lines (A549 and NCI-H1299) were obtained from American Type Culture Collection (ATCC). The cells were cultured in Dulbecco's Modified Eagle's Medium (DMEM, Hyclone, USA) with 10% fetal bovine serum (FBS, Hyclone, USA) at 37°C in a humidified atmosphere with 5% CO_2_. The control vectors, pGL3-TWIST2-wt, pGL3-TWIST2-mut, pcDNA3.1-TWIST2, shTWIST2, miR-22-5p inhibitors, and miR-22-5p mimics, were either obtained from Obio Technology (Shanghai, China) or GeneCopoeia (Guangzhou, China) or some of them were constructed by our group. Cell transfections were performed using transfection reagents (Thermo Scientific, USA) according to the manufacturer's instructions.

### 2.3. qRT-PCR

Total RNA from NSCLC cells was isolated using the TRlzol Reagent (Beyotime, China). Reverse transcription was achieved with the Quantscript RT Kit (Tiangen, China). SYBR RT-PCR Kit (Tiangen, China) was used to perform the transcript quantification with specific primers. Expression levels were quantified through the 2^−*ΔΔ*CT^ method with *GAPDH* as the reference gene. The reverse primer for RT-PCR was provided with the Quantscript RT Kit. The forward primer sequences of miR-22-5p and TWIST2 are as follows: miR-22-5p: 5′-AGTTCTTCAGTGGCAAGCTTTAAA-3′ and TWIST2: 5′-CAAGATCCAGACGCTCAAGCT-3′. The reverse primer for qPCR was provided with the SYBR RT-PCR Kit.

### 2.4. Cell Proliferation

Forty-eight-well culture plates were used for cell plating at a density of 5 × 10^3^ cells per well. The cells were collected with trypsin and resuspended in phosphate buffer (PBS) every 24 h for 4 days. The resuspended cells were counted with a light microscope (Nikon, Japan). Each experiment was performed in duplicate, and the results were presented in mean ± standard deviation (S.D.).

### 2.5. Wound Healing Assay

Twenty-four-well culture plates were used for cell plating at a density of 5 × 10^5^ cells per well. When the NSCLC cells adhered to the plate, a 200 *μ*L pipette tip was used to scratch a wound, and the incubation was continued in serum-free medium for another 48 h. Wound healing was observed with a light microscope (Nikon, Japan). Each experiment was performed in duplicate, and mean ± S.D. values were presented.

### 2.6. Matrigel Invasion Assay

The different treated NSCLC cells were seeded into the transwell cell culture inserts (Corning, USA) precoated with Matrigel (BD, USA). The NSCLC cells were cultured in serum-free medium on the upper chamber and serum on the lower chamber. Invasion from the upper chamber to the lower chamber was allowed for 48 h. The passed cells from the upper chamber were fixed in 4% paraformaldehyde, then stained with crystal violet solution, and observed with a light microscope (Nikon, Japan). Each experiment was performed in duplicate, and mean ± S.D. values were presented.

### 2.7. Western Blot Analysis

The total proteins in NSCLC cells were isolated by the RIPA lysate buffer (KeyGen, China). Western blot analysis was conducted by using the primary antibodies for TWIST2, E-cadherin, vimentin, and GAPDH (Affinity, USA), followed by secondary antibody (Affinity, USA). Blots were detected by using the enhanced chemiluminescence detection kit (Millipore, USA). Protein expression levels were analyzed using the ImageJ software. Each experiment was performed in duplicate, and mean ± S.D. values were presented.

### 2.8. Luciferase Reporter Assay

The luciferase reporter plasmids of pGL3-TWIST2-wt and pGL3-TWIST2-mut contained the 3′-untranslated regions (UTRs) of “5′-GCAAUGGCUAAGAACAU-3′” and “5′-GCAAUGGCUCAACUAAU-3′,” respectively. The reporter gene plasmids and miR-22-5p mimics were cotransfected into A549 cells, and culturing was continued for another 48 h. Luciferase activities were detected through the Dual-Luciferase Assay System (Promega) and tested with a Luminoskan Ascent Reader System (Thermo Scientific, USA).

### 2.9. Animal Studies

Twelve BALB/c nu/nu mice were randomly divided into four groups. Differently treated A549 cells were subcutaneously injected into the right flank of each mouse correspondingly at a density of 5 × 10^6^ cells/100 *μ*L/mouse. When the xenograft tumors grew to about grain size after 8 days, the tumor sizes were measured every 2 days for another 18 days. On the last day, all the mice were sacrificed, and xenograft tumors were collected.

### 2.10. Statistical Analysis

After normality and equal variance were tested across the groups, differences between groups were assessed using Student's *t*-test. All experiments were repeated at least three times. All results were shown as means ± S.D., and *P* < 0.05 was defined of statistically significant.

## 3. Results

### 3.1. Selection of Candidate miRNA in Regulating NSCLC Malignant Progression

**Figure 1:**
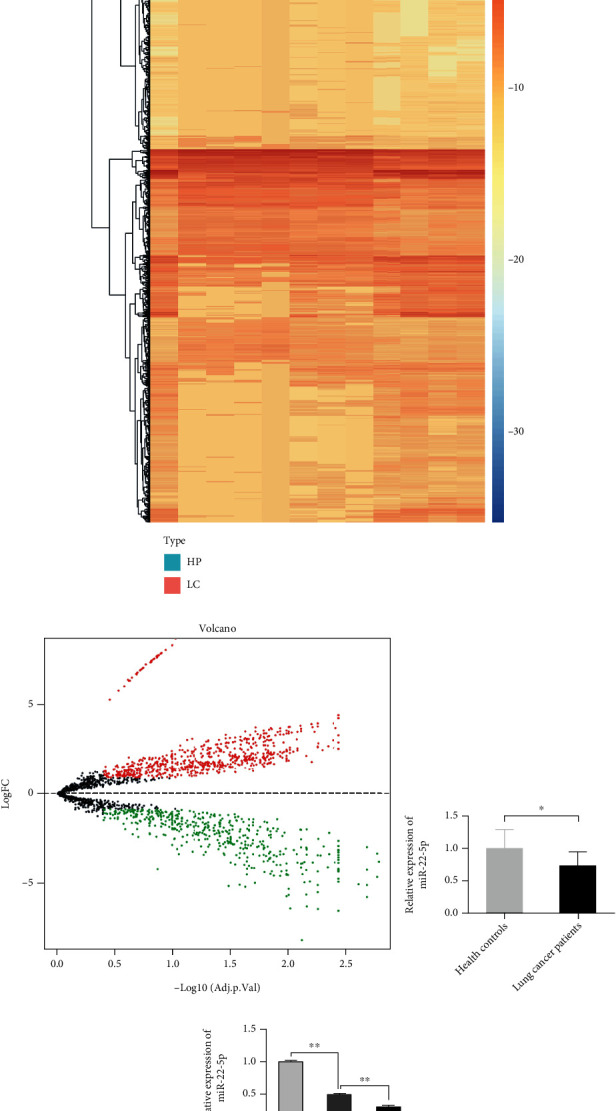
miR-22-5p is downregulated in NSCLC. (a) Heat map analysis for the comparison of miRNA expression profiles between NSCLC tissues and adjacent normal tissues. (b) Volcano plot of differentially expressed miRNAs between NSCLC tissues and adjacent normal tissues. (c) Relative expression of miR-22-5p in NSCLC tissues and adjacent normal tissues. (d) Relative expression of miR-22-5p in normal lung cells and NSCLC cells (mean ± S.D.; *n* = 3 in triplicate; ^∗∗^*P* < 0.01).

The miRNA expression profiles downloaded from the GEO database were used for identifying the candidate miRNA in regulating NSCLC malignant progression. The heat map results showed that the miRNA expression profiles between adjacent normal tissues and NSCLC tissues were very different (Figure 1(a)). The volcano plots further exhibited a dysregulated miRNA expression profiles between the two groups (Figure 1(b)). miR-22-5p was remarkably downregulated in NSCLC tissues than in adjacent normal tissues (Figure 1(c)). Three normal lung cells or NSCLC cells were selected to detect the expression levels of miR-22-5p and further demonstrate this trend. The qRT-PCR results showed that the miR-22-5p levels in NSCLC cells remarkably decreased compared with those in normal lung cells, and A549 cells had a substantially lower miR-22-5p level than NCI-H1299 cells (Figure 1(d)).

**Figure 2:**
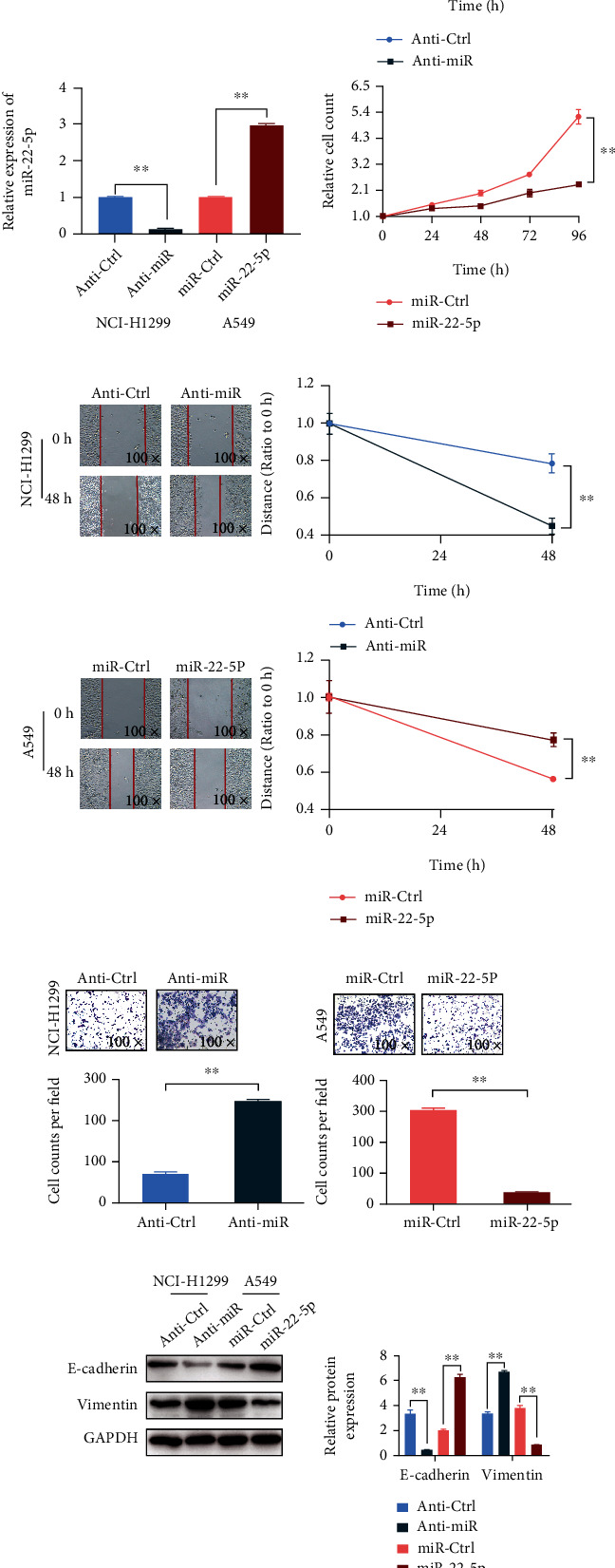
miR-22-5p regulates NSCLC cell proliferation, migration, invasion, and EMT. (a) miR-22-5p inhibitor could inhibit the content of miR-22-5p in NCI-H1299 cells, and miR-22-5p mimics could increase the content of miR-22-5p in A549 cells. (b) miR-22-5p downregulation promoted the proliferation of NCI-H1299 cells, and miR-22-5p upregulation inhibited the proliferation of A549 cells. (c) miR-22-5p inhibitor promoted the wound healing speed of NCI-H1299 cells, and miR-22-5p upregulation decreased the migration ability of A549 cells. (d) miR-22-5p inhibitor promoted the invasion of NCI-H1299 cells, and miR-22-5p upregulation decreased the invasion ability of A549 cells. (e) miR-22-5p inhibitor promoted vimentin expression and inhibited E-cadherin expression in NCI-H1299 cells, whereas miR-22-5p upregulation inhibited vimentin expression and increased E-cadherin expression in A549 cells (mean ± S.D.; *n* = 3 in triplicate; ^∗∗^*P* < 0.01).

miR-22-5p mimic and inhibitor were constructed to study the role of miR-22-5p in NSCLC. The miR-22-5p level considerably decreased in NCI-H1299 cells after the transfection of the miR-22-5p inhibitor and remarkably increased in A549 cells after the transfection of the miR-22-5p mimics (Figure 2(a)). The proliferation results showed that miR-22-5p downregulation could increase the proliferation of NCI-H1299 cells, whereas miR-22-5p upregulation inhibited the proliferation of A549 cells (Figure 2(b)). The transfection of the miR-22-5p inhibitor promoted the wound healing speed in NCI-H1299 cells, whereas the transfection of the miR-22-5p mimics considerably inhibited the migration ability of A549 cells (Figure 2(c)). Similar to the effects on cell migration, miR-22-5p also caused substantial differences in the invasion abilities of the two NSCLC cell models. The miR-22-5p inhibitor could increase the contents of invading NCI-H1299 cells, whereas miR-22-5p mimics almost completely removed the invasion ability of A549 cells (Figure 2(d)). Next, we detected the EMT-related markers, as EMT is closely related to metastasis and malignant progression. The Western blot results showed that the epithelial marker E-cadherin was remarkably downregulated, and the mesenchymal marker vimentin was substantially upregulated by the miR-22-5p inhibitor in NCI-H1299 cells. On the contrary, miR-22-5p mimics considerably increased the expression of E-cadherin and inhibited the expression of vimentin in A549 cells (Figure 2(e)).

### 3.2. TWIST2 Is Negatively Regulated by miR-22-5p as a Direct Target

**Figure 3:**
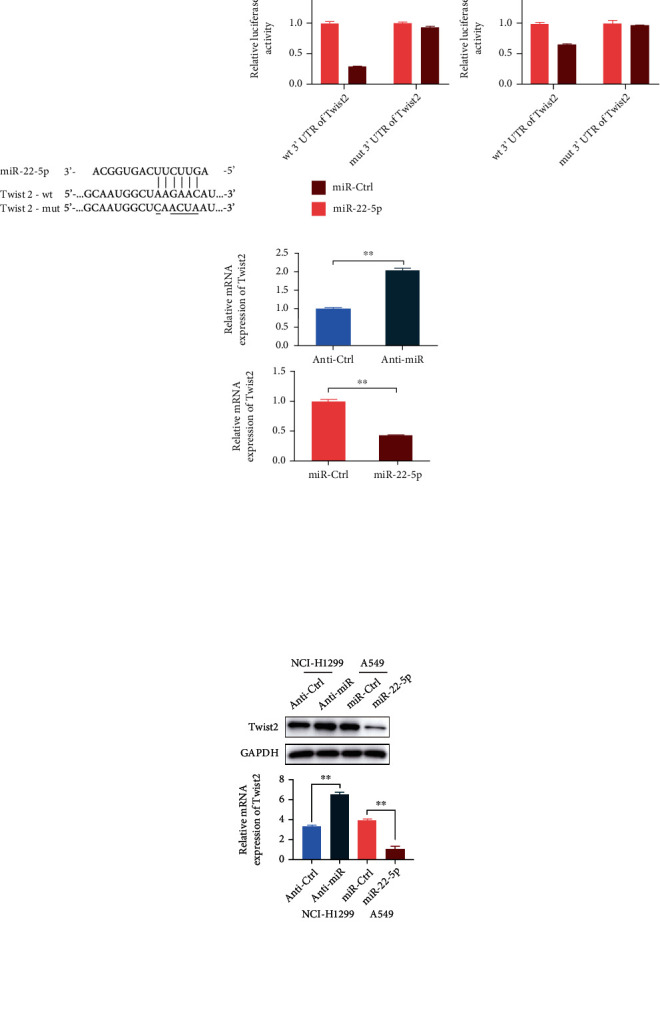
TWISTt2 is a direct target of miR-22-5p. (a) Schematic of the reporter construct of the wild-type and mutated TWIST2 3′-UTR sequences. (b) Luciferase reporter assay of the direct targeting activity of miR-22-5p to the TWIST2 3′-UTR by cotransfection with miR-22-5p mimics and wt/mut TWIST2 luciferase reporter vectors. wt: wild type; mut: mutated. (c) qRT-PCR analysis of the mRNA levels of TWIST2 regulated by miR-22-5p in NCI-H1299 and A549 cells. (d) Western blot analysis of the protein expression levels of TWIST2 regulated by miR-22-5p in NSCLC cells (mean ± S.D.; *n* = 3 in triplicate; ^∗∗^*P* < 0.01).

The potential target of miR-22-5p was detected using the TargetScan database to have a clearer understanding of the regulation mechanism of miR-22-5p in affecting the malignant progression of NSCLC. TWIST2 was considered as a putative target mRNA of miR-22-5p. This hypothesis was verified by constructing wild-type and mutated TWIST2 luciferase reporter vectors (Figure 3(a)). When the luciferase reporter vectors and miR-22-5p mimics were cotransfected into NCI-H1299 and A549 cells, a remarkable reduction in the luciferase activity of the wild-type TWIST2 reporter was found in both cells, whereas the mutated TWIST2 luciferase reporter had no effect on the inhibition of miR-22-5p (Figure 3(b)). When miR-22-5p inhibitor was transfected into NCI-H1299 cells, the mRNA content of TWIST2 markedly increased compared with that in the control group, and the TWIST2 mRNA expression was remarkably inhibited by miR-22-5p in A549 cells (Figure 3(c)). Western blot results further verified that miR-22-5p inhibitor substantially upregulated the TWIST2 protein expression, and the miR-22-5p mimics considerably downregulated the TWIST2 expression (Figure 3(d)).

### 3.3. miR-22-5p Relies on TWIST2 to Regulate NSCLC Cell Metastasis and EMT

**Figure 4:**
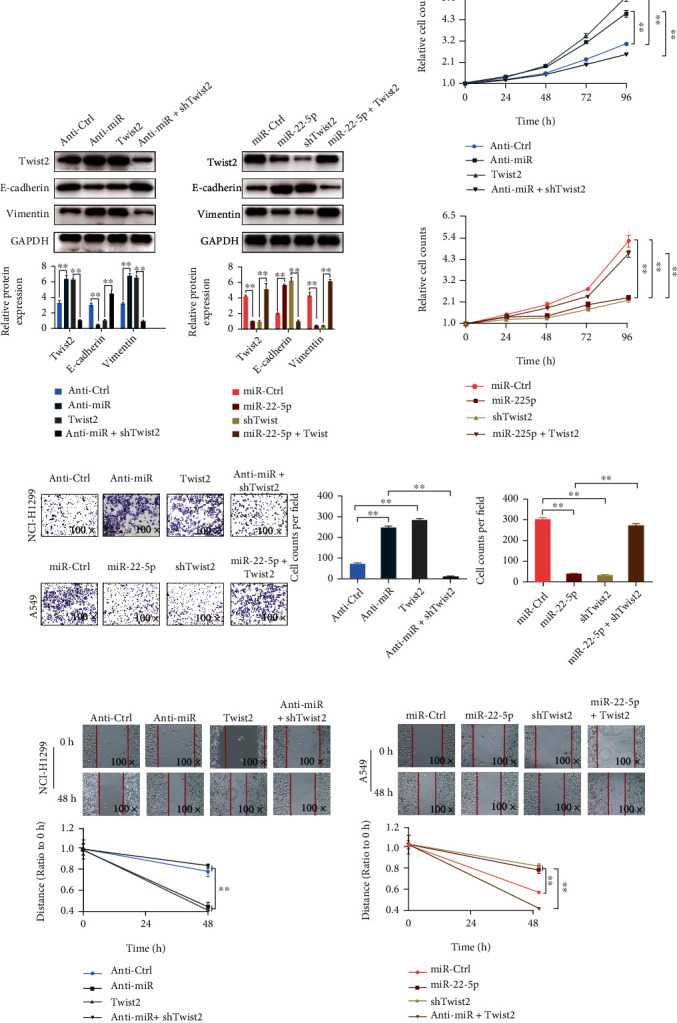
miR-22-5p relies on TWIST2 to regulate NSCLC cell EMT, proliferation, migration, and invasion. (a) Western blot analysis of the protein expression levels of TWIST2, E-cadherin, and vimentin regulated by miR-22-5p/TWIST2. (b) Effects of miR-22-5p/TWIST2 on the proliferation of NCI-H1299 and A549 cells. (c) Effects of miR-22-5p/TWIST2 on wound healing in NCI-H1299 and A549 cells. (d) Effects of miR-22-5p/TWIST2 on the invasion abilities of NCI-H1299 and A549 cells (mean ± S.D.; *n* = 3 in triplicate; ^∗∗^*P* < 0.01).

The effects of miR-22-5p/TWIST2 were studied in cell models. Western blot analysis results showed that TWIST2 and vimentin were remarkably upregulated when transfected with miR-22-5p inhibitor or pcDNA3.1-TWIST2 in NCI-H1299 cells, whereas E-cadherin was remarkably inhibited; TWIST2 knockdown could offset the effects of the miR-22-5p inhibitor on the upregulation of TWIST2 and vimentin and the downregulation of E-cadherin. TWIST2 upregulation could substantially alleviate the inhibition effects of miR-22-5p on TWIST2 and vimentin expression in A549 cells, and the E-cadherin expression decreased to a level similar to the control (Figure 4(a)). TWIST2 upregulation had a similar effect on cell proliferation with the transfection of the miR-22-5p inhibitor, but the proliferation ability remarkably increased when TWIST2 was knocked down even with the transfection of the miR-22-5p inhibitor. Cell proliferation ability remarkably decreased when the miR-22-5p mimics were added or TWIST2 was downregulated; however, the inhibition effect of miR-22-5p on proliferation ability could be offset to the control level by the upregulation of TWIST2 (Figure 4(b)). The wound healing assay and Matrigel invasion assay results showed a consistent trend with proliferation ability. The transfection of the miR-22-5p inhibitor or pcDNA3.1-TWIST2 could remarkably promote the wound healing speed and cell invasion ability of NCI-H1299 cells, whereas the transfection of the miR-22-5p mimics or shTWIST2 decreased the invasion and migration abilities of A549 cells. The wound healing speed and invasion ability of NCI-H1299 cells almost decreased to the control level when TWIST2 was knocked down, and the miR-22-5p inhibitor was added simultaneously. Migration and invasion abilities were remarkably enhanced when TWIST2 was upregulated by adding miR-22-5p mimics (Figure 4(d)).

**Figure 5:**
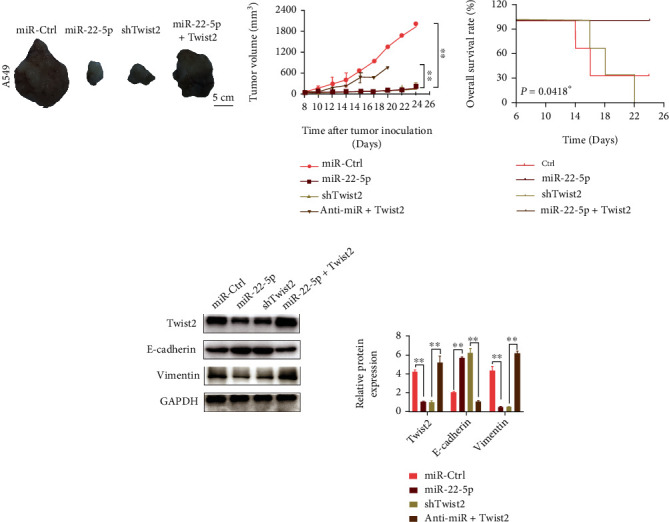
Effects of miR-22-5p/TWIST2 on NSCLC malignant progression in the A549 xenograft model. (a) miR-22-5p upregulation or TWIST2 knockdown inhibited A549 xenograft growth, whereas the TWIST2 overexpression offsets the inhibition effect of miR-22-5p. (b) Statistical analysis of the overall survival rate in different treated A549 xenograft models. (c) Western blot analysis of the protein expressions of TWIST2, E-cadherin, and vimentin in different treated xenograft tissues regulated by miR-22-5p/TWIST2 (mean ± S.D.; *n* = 3 in triplicate; ^∗∗^*P* < 0.01).

An in vivo A549 xenograft model was constructed to further illustrate the effects of miR-22-5p/TWIST2 on NSCLC. miR-22-5p upregulation or TWIST2 downregulation substantially decreased the tumor proliferation ability of NSCLC cells, whereas TWIST2 upregulation remarkably restored the proliferation ability damaged by miR-22-5p (Figure 5(a)). The survival rate analysis results showed an increase in NSCLC cell mortality after the overexpression of miR-22-5p and TWIST2. By contrast, the transfection of miR-22-5p or shTWIST2 considerably protected the mice from death by A549 xenograft tumors (Figure 5(b)). The tumor tissues were used to detect the expression levels of TWIST2, E-cadherin, and vimentin. Consistent with the in vitro experiment results, the addition of miR-22-5p or the knockdown of TWIST2 remarkably inhibited the expression of TWIST2 and vimentin and increased the expression of E-cadherin, and the upregulation of TWIST2 could offset the inhibition effect of miR-22-5p on TWIST2 and vimentin and the promotion effect on E-cadherin (Figure 5(c)).

## 4. Discussion

Cancer ranks as the first or second leading cause of death before the age of 70 years, and the burden of cancer incidence and mortality is rapidly growing worldwide [17]. Among difference cancers, the incidence of lung cancer ranks as the second in all 19.3 million new cases, and the mortality of lung cancer ranks as the first in all the 9.9 million deaths caused by cancer [1]. NSCLC accounts for more than 80% of lung cancer cases with a low 5-year survival rate [2]. Although a series of clinical treatments, including targeted therapy, chemotherapy, and immunotherapy, have been used for NSCLC, the mortality rate of NSCLC is still very high [4]. In addition to the fact that most patients with NSCLC are in the advanced stage, the lack of a more effective treatment is the main reason for the high mortality of NSCLC. Thus, a better understanding of the molecular mechanism of the malignant progression of NSCLC will provide more possibilities for the clinical treatment of NSCLC.

In this study, we aimed to identify a miRNA that regulates the malignant progression of NSCLC. miRNAs are small noncoding RNAs that recognize and bind to the 3′-UTR sites of the target genes and inducing the cleavage of mRNA or the inhibition of translation. miRNAs are divided into 3p and 5p, which are derived from the 3′ and 5′ ends of the hairpin of pre-miRNA, respectively; both forms could play a regulatory role [18, 19]. An increasing number of research demonstrated that miRNAs are involved in multiple cancers, but its effect is not enough because noncoding RNAs account for 98% of the entire genome [20]. Hence, further research on miRNAs that regulate NSCLC is needed to gain a clearer understanding of the mechanism.

**Figure 6:**
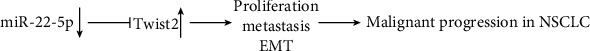
Regulatory mechanism of miR-22-5p during NSCLC malignant progression.

miR-22-5p was the miRNA we identified based on the analysis of the GEO database and our previous research [21]. miR-22-5p is a member of the miR-22 family and is located on chromosome 17p13.3. It has active regulatory effects in a series of diseases, such as acute myocardial infarction, Hashimoto's disease [22–24], and multiple tumors, by targeting the vascular endothelial factor, intercellular adhesion molecule 1, or Ras-association proximate 1/extracellular-signal regulated kinase signaling pathways [25–27]. In the present study, a series of target genes of miR-22-5p were predicted, as miR-22-5p could regulate the migration, proliferation, and EMT, the target of miR-22-5p must be associated to tumor malignant progression, and then we clarified that miR-22-5p could directly target TWIST2, a transcription factor associated with tumor malignant progression [28]. The in vitro and in vivo experiment results verified that miR-22-5p relied on TWIST2 to regulate the proliferation, metastasis, and EMT of NSCLC (Figure 6). miR-22 was also reported that could target Twist1 [29], as TWIST1 and TWIST2 both belong to the HLH structure family, and the genes with HLH structure might be the potential targets of miR-22. However, the target sequences of miR-22 in Twist1 were different with those of miR-22-5p in TWIST2, whether the genes with HLH structure are target genes of miR-22 needs for further confirmation. These results demonstrated that miR-22-5p is a tumor suppressor gene. Although a similar downregulation in miR-22-5p was found in hepatocellular carcinoma and breast cancer, miR-22-5p also has a tumor-promoting effect in prostate cancer [11, 13, 14, 30]. The controversial roles of miR-22-5p in tumor malignant progression might be influenced by different microenvironments, and the dual-directional regulatory activity of miR-22-5p needs further research.

Some research showed that TWIST2 could also regulate the expression of miR-22-5p [31]; thus, miR-22-5p and TWIST2 could form a direct feedback regulation loop. However, whether the miR-22-5p/TWIST2 and TWIST2/miR-22-5p regulation patterns play the leading role and how gene and protein expression levels are regulated are currently unclear. The different proteins interacting with TWIST2 might lead to a completely opposite mode of regulation; hence, this hypothesis needs further validation.

In summary, our results revealed that miR-22-5p was downregulated in NSCLC in a manner that is correlated with enhanced malignant progression. This study confirmed that miR-22-5p relied on TWIST2 to have an important active role in regulating the proliferation, metastasis, and EMT of NSCLC cells. Therefore, miR-22-5p could be a therapeutic target for the clinical treatment of NSCLC.

## Data Availability

The datasets used and/or analyzed in the present study are available from the corresponding author upon reasonable request.

## References

[1] Sung H., Ferlay J., Siegel R. L. (2021). Global cancer statistics 2020: GLOBOCAN estimates of incidence and mortality worldwide for 36 cancers in 185 countries. *CA: a Cancer Journal for Clinicians*.

[2] Ye Z., Huang Y., Ke J., Zhu X., Leng S., Luo H. (2021). Breakthrough in targeted therapy for non-small cell lung cancer. *Biomedicine & Pharmacotherapy = Biomedecine & Pharmacotherapie*.

[3] Schrank Z., Chhabra G., Lin L. (2018). Current molecular-targeted therapies in NSCLC and their mechanism of resistance. *Cancers*.

[4] Carmichael J. A., Wing-San Mak D., O'Brien M. (2018). A review of recent advances in the treatment of elderly and poor performance NSCLC. *Cancers*.

[5] Bylicki O., Barazzutti H., Paleiron N., Margery J., Assie J. B., Chouaid C. (2019). First-line treatment of non-small-cell lung cancer (NSCLC) with immune checkpoint inhibitors. *BioDrugs*.

[6] Wittekind C., Neid M. (2005). Cancer invasion and metastasis. *Oncology*.

[7] Nieto M. A., Huang R. Y., Jackson R. A., Thiery J. P. (2016). Emt: 2016. *Cell*.

[8] Amorim M., Salta S., Henrique R., Jeronimo C. (2016). Decoding the usefulness of non-coding RNAs as breast cancer markers. *Journal of Translational Medicine*.

[9] Zhao L., Liu W., Xiao J., Cao B. (2015). The role of exosomes and "exosomal shuttle micro RNA" in tumorigenesis and drug resistance. *Cancer Letters*.

[10] Reddy K. B., Micro R. N. A. (2015). MicroRNA (miRNA) in cancer. *Cancer Cell International*.

[11] Jin Q., Hu H., Yan S. (2021). lncRNA MIR22HG-derived miR-22-5p enhances the radiosensitivity of hepatocellular carcinoma by increasing histone acetylation through the inhibition of HDAC2 activity. *Frontiers in Oncology*.

[12] Wang J., Zhang H., Zhou X. (2018). Five serum-based miRNAs were identified as potential diagnostic biomarkers in gastric cardia adenocarcinoma. *Cancer Biomarkers: Section A of Disease Markers*.

[13] Wang D., Guo C., Kong T., Mi G., Li J., Sun Y. (2019). Serum miR-22 may be a biomarker for papillary thyroid cancer. *Oncology Letters*.

[14] Wang B., Li D., Filkowski J. (2018). A dual role of miR-22 modulated by RelA/p65 in resensitizing fulvestrant- resistant breast cancer cells to fulvestrant by targeting _FOXP1_ and _HDAC4_ and constitutive acetylation of p53 at Lys382. *Oncogene*.

[15] Ritchie M. E., Phipson B., Wu D. (2015). Limma powers differential expression analyses for RNA-sequencing and microarray studies. *Nucleic Acids Research*.

[16] Agarwal V., Bell G. W., Nam J. W., Bartel D. P. (2015). Predicting effective microRNA target sites in mammalian mRNAs. *eLife*.

[17] Siegel R. L., Miller K. D., Jemal A. (2020). Cancer statistics, 2020. *CA: a Cancer Journal for Clinicians*.

[18] Park J. E., Heo I., Tian Y. (2011). Dicer recognizes the 5′ end of RNA for efficient and accurate processing. *Nature*.

[19] Lund E., Dahlberg J. E. (2006). Substrate selectivity of exportin 5 and dicer in the biogenesis of microRNAs. *Cold Spring Harbor Symposia on Quantitative Biology*.

[20] Mattick J. S. (2001). Non-coding RNAs: the architects of eukaryotic complexity. *EMBO Reports*.

[21] Jiang W., Han X., Wang J. (2019). miR-22 enhances the radiosensitivity of small-cell lung cancer by targeting the WRNIP1. *Journal of Cellular Biochemistry*.

[22] Li H., Zhang P., Li F. (2019). Plasma miR-22-5p, miR-132-5p, and miR-150-3p are associated with acute myocardial infarction. *Biomed Research International*.

[23] Trummer O., Foessl I., Schweighofer N. (2022). Expression profiles of miR-22-5p and miR-142-3p indicate Hashimoto’s disease and are related to thyroid antibodies. *Genes*.

[24] Li Y., Liu H., Cui Y. (2020). miR-424-3p contributes to the malignant progression and chemoresistance of gastric cancer. *OncoTargets and Therapy*.

[25] Yang X., Zhang Y., Li Y., Wen T. (2018). MALAT1 enhanced the proliferation of human osteoblasts treated with ultra‑high molecular weight polyethylene by targeting VEGF via miR‑22‑5p. *International Journal of Molecular Medicine*.

[26] Gidlof O., Sathanoori R., Magistri M. (2015). Extracellular uridine triphosphate and adenosine triphosphate attenuate endothelial inflammation through miR-22-mediated ICAM-1 inhibition. *Journal of Vascular Research*.

[27] Qin M., Li Q., Wang Y. (2021). Rutin treats myocardial damage caused by pirarubicin via regulating miR-22-5p-regulated RAP1/ERK signaling pathway. *Journal of Biochemical and Molecular Toxicology*.

[28] Li S., Chen K., Zhang Y. (2019). TWIST2 amplification in rhabdomyosarcoma represses myogenesis and promotes oncogenesis by redirecting MyoD DNA binding. *Genes & Development*.

[29] Zhu S. T., Wang X., Wang J. Y., Xi G. H., Liu Y. (2020). Downregulation of miR-22 contributes to epithelial-mesenchymal transition in osteosarcoma by targeting Twist1. *Frontiers in Oncology*.

[30] Zhang J., Yang Y., Yang T. (2010). MicroRNA-22, downregulated in hepatocellular carcinoma and correlated with prognosis, suppresses cell proliferation and tumourigenicity. *British Journal of Cancer*.

[31] Vesuna F., Lisok A., van Diest P., Raman V. (2021). Twist activates miR-22 to suppress estrogen receptor alpha in breast cancer. *Molecular and Cellular Biochemistry*.

